# Modelling Informal Carers’ Health-Related Quality of Life: Challenges for Economic Evaluation

**DOI:** 10.1007/s40258-023-00834-4

**Published:** 2023-11-10

**Authors:** Becky Pennington, Hareth Al-Janabi

**Affiliations:** 1 Sheffield Centre for Health and Related Research (SCHARR), School of Medicine and Population Health, University of Sheffield, Sheffield, UK; 2 Health Economics Unit, Institute of Applied Health Research, University of Birmingham, Birmingham, UK

## Abstract

There has been increasing interest in including carers’ health-related qualify of life (HRQoL) in decision models, but currently there is no best practice guidance as to how to do so. Models thus far have typically assumed that carers’ HRQoL can be predicted from patient health states, as we illustrate with three examples of disease-modifying treatments. However, this approach limits the mechanisms that influence carers’ HRQoL solely to patient health and may not accurately reflect carers’ outcomes. In this article, we identify and discuss challenges associated with modelling intervention effects on carers’ HRQoL: attaching carer utilities to patient disease states, the size of the caring network, aggregation of carer and patient HRQoL, patient death, and modelling longer-term carer HRQoL. We review and critique potential alternatives to modelling carers’ HRQoL in decision models: trial-based analyses, qualitative consideration, cost-consequence analysis, and multicriteria decision analysis, noting that each of these also has its own challenges. We provide a framework of issues to consider when modelling carers’ HRQoL and suggest how these can be addressed in current practice and future research.

## Key Points for Decision Makers

**Table:** 

Economic evaluations typically assume that carers’ health-related quality of life (HRQoL) can be predicted from patient health states, but interventions may affect carers’ HRQoL in other ways.
There are also challenges in considering the number of carers, aggregating patient and carer HRQoL, patient death and longer-term extrapolation.
Current approaches rely on cross-sectional data, but longitudinal analysis is needed to show how carers’ HRQoL changes over time.

## Introduction

There has been an increasing interest in the inclusion of family/informal carers’ health-related quality of life (HRQoL) [or ‘spillovers’] in economic evaluations [1–4], with a number of health technology assessment (HTA) bodies recognising their importance [5–7]. Indeed, the inclusion of carers’ HRQoL in appraisals by the National Institute for Health and Care Excellence (NICE) has increased from 2.9% of Technology Appraisals (TAs) and 50% of Highly Specialised Technologies (HSTs) in 2000–2019 [8] to 5.7% and 78%, respectively, in 2019–2022 [9].

Current guidance may recommend that carers are included [5, 10] or make recommendations relating to justification and transparency [7, 11] or for future research [12], but there is currently little guidance as to *how* HRQoL for carers should be included in economic evaluation (other than to state results including carers should be presented separately) [13].

The inclusion of carers in economic models to estimate the cost effectiveness of patient interventions has the potential to change results and reimbursement decisions [7, 14]. Furthermore, different methods to model carer’s HRQoL may lead to different results (such as the use of carer utilities or disutilities and the inclusion of carer HRQoL beyond patient death [15]) and also have different implications for how carers are valued. In this paper, we provide an overview of the current commonly employed techniques, discuss the challenges associated with them, and suggest key areas for consideration. We hope that illuminating these issues will encourage debate and the development of methods for including informal carers’ HRQoL in economic evaluation. We provide a framework for addressing these issues in current practice and future research.

## Current Approach to Modelling Carers’ Health-Related Quality of Life (HRQoL) in Decision Models

In economic evaluation, decision models may be used to estimate the costs and outcomes of an intervention and its comparator(s) over the required time horizon (typically longer than the associated clinical trials). They can combine multiple sources of evidence and can incorporate uncertainty associated with the structure and parameters used to populate them. Decision models represent a useful tool to help decision makers understand the long-term costs and effects of an intervention. In cost-utility analysis for healthcare interventions, the costs may be limited to those accrued by the healthcare sector (for example, NICE in England and Wales [5]) or may also include costs to patients and other sectors (for example, the Zorginstituut in The Netherlands [10]). Outcomes are typically health effects expressed in quality-adjusted life-years (QALYs), calculated through estimating changes to length of life (through survival/mortality) and quality of life (through the use of utilities). Traditionally, models have focused on interventions targeted at patients and therefore the outcomes have, perhaps understandably, been limited to patient QALYs. However, interventions for patients may also affect their informal carers [16], and therefore the need to consider both patients and family carers has been increasingly recognised, as reflected in the growing literature around carer utilities [2].

In decision models, the patient’s health trajectory over the time horizon can be estimated from multiple data sources with utilities attached to different health states to represent HRQoL. This may require evidence beyond clinical trials, including observational and registry studies [17]. Here, we consider three examples of economic models for disease-modifying therapies (DMTs) where carers’ HRQoL has also been included: multiple sclerosis (MS), Alzheimer’s disease, and spinal muscular atrophy (SMA). In each case, specific datasets and mathematical approaches were employed to simulate the patients’ HRQoL trajectory over the lifetime.

- In economic models of MS, the natural history for disability progression, relapse rates and mortality may be derived from longitudinal datasets, surveys and registries, with the relative treatment effectiveness applied calculated from randomised controlled trials (RCTs). Each modelled health state (defined by the Kurtzke Extended Disability Status Scale [EDSS]) has a patient utility (anchored between 0 and 1, with worse health states generally having lower utilities, and utilities with negative values representing health states worse than death) based on survey data, and relapses are associated with disutilities (1 minus the utility) [18].
- National datasets have similarly been used to inform health state transitions and mortality in economic models for Alzheimer’s disease, with relative effectiveness calculated from trials and patient utilities assigned to the health states [19].
- In an economic model for SMA, transitions between health states beyond the trial follow-up were estimated based on the rate of motor function score change within the trial, assuming that patients receiving the intervention continued to improve whereas patients receiving standard of care continued to worsen. Mortality within the trial period was estimated using parametric survival functions fitted to observed data, and beyond the trial period a function was fitted to survival data from an external long-term data source. Utilities were assigned to the health states based on a vignette study [20].

In each of these examples, carers’ HRQoL was modelled by attaching a carer utility or disutility (1 minus the utility) to the patient's health state. In the MS example, carer disutilities ranged from 0 (for the best patient health states) to 0.14 for the most progressed disease state, based on utilities reported for carers of patients with Alzheimer’s disease (the authors noted that actual measurements for MS were not available). The ‘total’ utility for each health state was calculated by subtracting the carer disutility from the patient utility, to give a utility that is lower than the original patient utility (disutilities for other patient factors may also be subtracted; for example, relapses and adverse events). This assumes that there is one patient and one carer, that each are equally valued, and that carers’ HRQoL is included until the patient died. The examples of DMTs in Alzheimer’s disease and SMA followed the same approach, with maximum carer disutilities of 0.10 for Alzheimer’s disease [19] and 0.16 for SMA [20].

A variation on this approach (included in some appraisals considered by NICE, the Tandvårds-och läkemedelsförmånsverket in Sweden and the Zorginstituut [7, 10]) is to include carer utilities (anchored between 0 and 1, like patient utilities) rather than disutilities. In this case, the patient and carer utilities are effectively added together, to give a number anchored between 0 and 2, although patient and carer QALYs are typically calculated and reported separately (and then summed). Mathematically, these two approaches are equivalent. The difference arises depending on whether carer utilities are included after the patient dies.

## Challenges

### Attaching Carer Utilities to Patient Disease States

This is the most common approach in decision models to date (including the three mentioned earlier) [7, 14] and may seem like a logical next step in extending the economic model to additionally include carers, as it requires relatively little data or additional estimation techniques. However, economic evaluations aim to evaluate the impact of the intervention on (patient and) carer health, not the impact of patient illness on (patient and) carer health, and attaching carer utilities to patient health status in this way implies the only pathway by which the intervention affects carers is via impact on the patient health status. In reality, this is only one of several possible pathways [21] and therefore risks not accurately reflecting the carer’s experience and may under- or overestimate the effect of interventions on carer’s HRQoL (see Sect. 3.5).

Carers' HRQoL may be considered as a function of both patients' health (the family effect) and caregiving burden (the caring effect) [22, 23]. Clearly, a relationship between patients' and carers' HRQoL exists and using such published relationships may provide a relatively simple method for estimating carers' QALYs without the need to model carers with the same granularity as patients. However, the existence of the caring effect demonstrates that act of providing care is also a significant predictor of carers' HRQoL, and therefore, at the very least, the caregiving burden should be considered in addition to patient health status when using published relationships, particularly where disease areas, interventions and contexts differ [22, 23].

A key limitation of much of the existing literature studying the family and caring effects lie in the cross-sectional study design (although we note some analyses have considered changes in patients' and carers’ HRQoL [24]). Authors discuss this limitation themselves, noting that they could not correct for baseline health before caregiving/patient ill health, and that future studies should use longitudinal datasets to address the causality of relationships [23]. While cross-sectional studies can identify between-people differences for different levels of caregiving, they do not identify within-people differences (the causal effect of caregiving). Using cross-sectional data (without casual inference methods) to model changes in carers’ HRQoL over time is therefore inaccurate and may lead to under- or overestimation of the changes in carer’s HRQoL.

### Number of Carers

Although many economic evaluations typically consider only one carer [7, 8] per patient, there is evidence that the impacts of caring can spill over onto multiple people; for example, an average of eight individuals for end of life care [25]. In the context of either a clinical trial or an observational study, collecting HRQoL data from each affected person may not be practical or feasible. There is therefore a need to consider how to model HRQoL effects for this caring/family network.

As an example, the NICE committee for the appraisal of nusinersen for SMA concluded that SMA has a substantial effect on the quality of life of carers and family members, and noted the difficulties in including carers' HRQoL. Patient experts described the impact of SMA on the extended family, including grandparents, siblings, and family friends, as well as the support required from parents or carers. The economic analysis included carer utilities for two to three carers (depending on health state) [26].

We may expect that some patient interventions have an impact on multiple family members, but that the size of the impact may vary between the people in this network. There is therefore a judgement required in determining how many carers should be included and whether the same HRQoL impact applies to all carers. In a regression analysis, Al-Janabi et al. found a positive spillover coefficient of 0.16 for the effect of patient EQ-5D on the health status of family members exposed to after-effects of meningitis [27]. If assuming a constant impact across the family network, they proposed that the total health benefits from the intervention for economic evaluation could be estimated by multiplying any QALY gains generated by patient HRQoL improvement by 1 + (*n* * 0.16), where *n* is the number of close family members. Analysts could use such a ‘multiplier’ (estimated from their own data as appropriate) to include multiple informal carers who all experience the same HRQoL impact.

As an alternative, Al-Janabi et al. also fitted a distance-decay function to project total spillovers beyond the closest family members—such a function can reflect the decreasing HRQoL impact with increasing social distance. Their multiplier of 1.48 was used in the Joint Committee for Vaccination and Immunisation’s appraisal of Bexsero [28] used to estimate the QALY loss to carers relative to the QALY loss of the patient.

This ‘multiplier’ may offer a framework for considering HRQoL of multiple carers in economic models, either to present the relationship between the relative QALY gains estimated within the study or to estimate the carers’ HRQoL changes where primary data are not available. Section 3.1 discussed the limitations associated with assuming that carer HRQoL is solely a function of patient HRQoL, therefore it may be not appropriate to transfer patient:carer QALY multipliers from different populations/interventions. However, they are likely to be useful for summarising and presenting carer QALYs alongside patient QALYs in a consistent and comprehensive way, as well as enabling the relative incremental cost-effectiveness ratios (ICERs) to be viewed, including and excluding spillovers. Predicting HRQoL effects for multiple carers may be desirable but ideally requires data on the size of the spillovers for the first and second closest family member. While this is clearly more feasible than collecting data for the entire network, it represents an additional burden compared with collecting data for the primary carer only. On the other hand, including data for the primary carer only either requires the assumption that the spillover effect is limited to one person or that it is consistent across the broader network and that the extent of the network is known.

The size of a person’s care network may also depend on other factors not necessarily related to the health condition or intervention (for example, the size of their household). Basu and Meltzer highlighted the potential equity concerns arising from considering varying numbers of family/household members when incorporating spillover effects, and suggested that weighted averages of family sizes (for example) and equity weights could be considered in both population-level and distributional cost-effectiveness analyses to avoid ethical concerns [29]. Such an approach requires even more data, both on the average family sizes for different health conditions and on the size of equity weights.

### Aggregation

The case studies discussed earlier in this paper all assumed that patient and carer QALYs can be summed to estimate total QALYs. This assumes that patient and carer QALYs are interchangeable; conceptually this may not be appropriate where different HRQoL measures were used to generate patient and carer utilities (for example, if the CarerQol-7D was used for carers and the EQ-5D for patients). An equal valuation of carer and patient QALYs may also not reflect public preferences (where considered by HTA agencies). For example, carers’ HRQoL effects were found to be valued at 74% of an equivalent effect on patient HRQoL [30].

Both the Zoorginstituut and, more recently, NICE, permit the application of equity weights for patients’ disease severity [5, 31] An additional (> 1) weighting is applied to the incremental QALY gain for the intervention if the QALY shortfall associated with the condition (calculated by comparing the remaining QALYs for a patient with the condition with those for the general population) is above a defined threshold. Although NICE state that the QALY shortfall calculations should not include carers, the use of severity weights within HTA demonstrate that there is a precedent for formally giving additional weight to interventions that meet some specific criteria. It is possible that a similar approach could be taken to derive equity weights for interventions or diseases with the highest burden to carers.

### Patient Death

Where economic models take a lifetime perspective (as is common), analysts and policy makers will need to decide whether the carer is included until the patient dies or until the carer dies. This may be particularly pertinent in the case where the carer is substantially younger or older than the patient, as in the case of children caring for parents or vice-versa. The issue of modelling carers’ HRQoL beyond the patients' lifetime has raised controversies in the choice of attaching either carer utilities or carer disutilities to patient health states. This is not because the two approaches are practically different but because in the historic disutility approach, carers' HRQoL was framed as an extension of patients’ HRQoL, with carer disutilities applied in the same way as disease relapses or adverse events. Including carer utilities instead represents carers as additional entities within the model, who may outlive the patient. An ethical judgement is therefore required to determine whether the carer should be included after the patient dies, noting that to do so may assume carer’s HRQoL improves after the patient dies (if the negative effect of bereavement on HRQoL is smaller than the negative effect of caring), but excluding carers after the patient dies has been criticised as it assumes that society places no value on the bereaved carer [15].

### Modelling Carers' HRQoL Trajectories

The methods discussed above focus on modelling patients first and then modelling carers in relation to this. However, independently modelling patients' and carers' health trajectories would allow consideration of how the intervention itself affects carers’ HRQoL directly, rather than indirectly via changes in patients’ health. Previous work describes six mechanisms by which healthcare delivery can influence carer’s wellbeing and patient outcomes is only one of these [21]. For example, transferring the responsibility of providing patient care between informal carers and formal services may have no impact on the patients' health status, but it would be inaccurate in this case to assume that because intervention had no impact on patients, it also had no impact on carers as the caregiving load for the family is likely to have significantly changed. Similarly, interventions that consider different staffing levels, information provision, or settings of delivery are likely to affect carer wellbeing independently of (as well as through) the patients' HRQoL.

Aside from assuming a simple utility increment for carers of patients receiving the intervention [32], we are unaware of any studies that have tried to estimate long-term health trajectories for carers from interventions, likely because of the complexities and data requirements (particularly longitudinal data) to do so. This approach would be analogous to measuring carers' HRQoL directly for the duration of a clinical trial and then extrapolating, and would likely require the same level of data as is required for modelling patients beyond the trial period. While this would represent an additional burden for analysts in terms of data collection and modelling, this is already a standard approach for modelling patients within clinical and economic evaluation. Furthermore, if we are serious that informal carers’ HRQoL should also be included, then it seems inequitable to neglect to do so with the same level of accuracy.

The longitudinal trajectories of informal carers’ health (in particular psychological/mental health) may be complex and subject to an ‘adaptation’ effect wherein the demands of caregiving are greatest upon starting caregiving [33] and/or a ‘wear and tear’ or ‘unexpected career’ effect wherein the hardships associated with caregiving accumulate gradually over time [34, 35]. Fully reflecting the longer-term effects of informal carers' HRQoL in an economic model is therefore challenging, especially due to the paucity of longitudinal data.

There may be aspects of informal carers’ HRQoL trajectories that are not patient disease-specific, which may be useful for informing models across multiple interventions and indications. Instead of focusing on how only patient health affects carers, it may be useful to consider the intensity/burden of informal caring provided, duration of caring, and transitions into and out of the caring role. A deeper understanding of what is important to carers and their HRQoL would also be informative to ensure models accurately reflect the effect of interventions.

The interactions between patients and carers represents an additional challenge when modelling their trajectories separately, recognising the relationship between patient and carer HRQoL as discussed in Sect. 3.1.

## Potential Alternatives to Modelling Carers' HRQoL

### Trial-Based Analyses

While still uncommon, the inclusion of carers’ HRQoL in economic evaluations alongside clinical trials is arguably more straightforward than in decision models—carers’ HRQoL is collected at various time points (like patient HRQoL) and QALYs are calculated over the duration of the trial [36]. Under this approach, carers and patients are treated as separate entities with their own individual and (potentially) unrelated HRQoL trajectories. Patient and carer QALYs may be summed or reported separately to allow decision makers to consider the aggregated or disaggregated benefits. For example, Lamb et al. used the area under the curve method and linear interpolation between the assessment points in an RCT for an exercise programme for people with mild to moderate dementia and reported 0.787 QALY for the patient and 0.758 for the carer for the intervention, and 0.826 for the patient and 0.782 for the carer for usual care (difference not significant at the 5% level) over 12 months [37]. Chatterton et al. calculated QALYs for participants and their parents at 12-month follow-up in a trial of the management of childhood anxiety disorders and reported 0.687 youth QALY and 0.785 parental QALY for the intervention, and 0.693 youth QALY and 0.792 parental QALY for the comparator (difference not significant at the 5% level) [38]. However, clinical trials are typically limited in duration and it is unlikely to be feasible to collect patient and carer data over the whole relevant time horizon, thus requiring some longer-term modelling and the associated challenges discussed earlier. Furthermore, economic evaluation alongside a single clinical trial is unlikely to be a sufficient basis for decision making [39].

### Qualitative Consideration

Qualitative consideration of the overall size of health benefits to (patients and) carers is used by NICE in their HST programme [5] and the effects on carers and families is included in the voting of the appraisal committee by the Institute for Clinical and Economic Review in the US [5, 40]. Such an approach allows decision makers to assign an additional ‘weight’ to interventions where there is anticipated to be an additional effect for carers, or to consider a higher cost-effectiveness threshold. This could involve considering personal testimony and/or qualitative data of carers at committee meetings alongside model outputs based on patient QALYs. However, without a formally defined process for the size of carers’ HRQoL and the weights or thresholds, qualitative consideration is not transparent, is not necessarily consistent across appraisals and may be open to bias.

### Cost-Consequences Analysis

Cost-consequence analysis lists all costs and outcomes separately. This may allow decision makers to take a broader perspective than cost-utility analysis, where a single ICER is presented [41], or to consider only specific outcomes and therefore may be useful especially for different decision makers who are interested in different items. Cost-consequence analysis would provide a clear breakdown of what the effects are to both patients and carers (as an example, see Grosse et al. [42]), but does not aggregate outcomes to determine the overall benefit of an intervention. This method still requires an estimation of what the effects are to carers, which may rely on the methods described in Sect. 3.

### Multicriteria Decision Analysis

Multicriteria decision analysis (MCDA) allows decisions makers to quantitatively combine multiple elements into one assessment [43], and may therefore represent a logical extension to combining qualitative consideration of carers’ HRQoL with cost-effectiveness analysis. MCDA has already been proposed in rare conditions [44] and frailty/complex conditions [45–47], two settings where carers’ HRQoL has been particularly common in cost-effectiveness analysis.

Methodological challenges have limited the use of MCDA in HTA to date [48, 49], but if it were routinely used within an HTA programme, it is feasible that carers’ HRQoL (alongside patients' HRQoL) could be included as a criterion for evaluating interventions and comparators. However, like cost-consequence analysis, this still requires estimation of the size of carers' HRQoL. Additionally, this would require the derivation of relative importance weights (an area where carers could be involved).

## A Framework for Modelling Informal Carers’ HRQoL

In Table 1, we propose a framework for modelling informal carers' HRQoL in economic evaluation. Given the challenges associated with current approaches to modelling informal carers’ HRQoL described in Sect. 3, and the limitations associated with the alternatives outlined in Sect. 4, we believe that such a framework is necessary and useful for informing current practice and future research. The framework lists specific issues that arise in modelling carers’ HRQoL and then provides examples of how these questions could be answered in both current practice (without collecting any new data) and future research (new data collection or analysis). The list of issues is not meant to be exhaustive but reflects the key issues arising in this paper. The examples provided are not intended to be prescriptive, but we hope they will aid understanding of how carers’ HRQoL can be modelled in current practice (for example, if economists/modellers complete a checklist based on this framework) and in inspiring future research to answer these questions.

**Table 1:** Suggestions for including carers' HRQoL in model-based economic evaluation

Issue	Suggestion for current practice	Suggestion for future research
Rationale for including carers' HRQoL	Explain the causal mechanism by which the intervention is anticipated to affect carers' HRQoL, supported by evidence. Justify the ‘transfer’ of utilities from other disease/intervention contexts	Develop methods for considering carers' HRQoL effects that do not arise as a result of changes in patients' HRQoL, such as regression modelling with other covariates and prospective collection from RCTs of data on intervention effects on carers
Time horizon of effect on carers' HRQoL	Explain assumptions about short-term (e.g., RCT period) and long-term effects of intervention on carers' HRQoL	Include carers’ HRQoL in registries and/or longitudinal analysis of observational datasets. Undertake longitudinal analyses to identify within-person effects
Size of caring network	Specify assumption about the size of the caring network and whether effect is consistent across carers within this network. Use a multiplier approach to synthesise this information into the ICER estimate	Where feasible, quantify how interventions affect the HRQoL of patients’ caring networks. Use this to generate estimates of the way in which spillovers spread with social distance to assist in calculating multipliers for future studies
Bereavement	Clarify what assumption is made about the impact of patient death on carers' HRQoL	Generate and, where relevant, incorporate estimates of HRQoL of patient death on carers’ HRQoL

*HRQoL* health-related quality of life, *ICER* incremental cost-effectiveness ratio, *RCT* randomised controlled trial

## Conclusion

Modelling is used in health economic evaluation to ensure the full range of costs and outcomes relevant to the decision problem are properly considered. In principle, the relevant outcomes in many cases include effects on carers’ as well as patients’ HRQoL. However, the treatment effect on carers is often unknown and therefore, typically, assumptions have been made about carers’ HRQoL and how it relates to patient health states. This article has discussed some of the key considerations when doing this and has highlighted the possibility of developing decision models that model carers’ HRQoL in a way that does not depend entirely on patient health states. We present a framework of issues to consider when modelling carers’ HRQoL. We hope this aids understanding of the challenges of including carers’ HRQoL in economic models and encourages transparency, consistency, and innovation in future analyses.

## Declarations

### Availability of data and materials

All information used in this manuscript can be found in the provided references.
